# Evolution or extinction? Paediatric and adolescent HIV responses in the Agenda 2030 era

**DOI:** 10.1002/jia2.25071

**Published:** 2018-02-27

**Authors:** Douglas Webb, Lucie Cluver, Chewe Luo

**Affiliations:** ^1^ HIV, Health and Development Group United Nations Development Programme (UNDP) New York NY USA; ^2^ Department of Social Policy and Intervention Oxford University Oxford United Kingdom; ^3^ Department of Psychiatry and Mental Health University of Cape Town Cape Town South Africa; ^4^ HIV Section Programme Division United Nations Fund for Children (UNICEF) New York NY USA

**Keywords:** Sustainable Development Goals, HIV, children, paediatric, adolescents

For the past 30 years, HIV has united the international community in an unprecedented fashion. Grassroot groups have mobilized in both the north and the south; policy‐makers and donors have worked with civil society, researchers and the private sector, and the epidemic response has inspired new thinking and medical innovation. The Millennium Development Goal (MDG) era was epitomized by the HIV response. For example, the remarkable progress on antiretroviral therapy has put the world closer to reaching the global target on reducing AIDS‐related deaths. Since 2000, two million HIV infections have been averted in children as a result of pregnant women living with HIV being able to access antiretroviral medicines 1. This progress has motivated the global HIV community to commit to fast track the response to end AIDS by 2030, a target in the new development era 2.

During this time, much of the advocacy for the importance of paediatric and adolescent HIV has been within the HIV/AIDS constituency itself, ensuring that children are not forgotten or discriminated against in the global HIV response 3. Advocacy has focused on prevention of mother to child transmission (PMTCT), meeting the specific needs and conditions of children living with HIV and advancing the complex interventions related to preventing infection in adolescents. Despite this, progress in ensuring access to antiretroviral treatment for children and adolescents has been slower than for pregnant women and adults 4, and we continue to witness the slow progress in preventing new infections in adolescents 5.

The Agenda 2030 and associated Sustainable Development Goals (SDGs) now bring a fundamental change. Instead of having a Millennium Development Goal focused on combating the major infectious diseases of HIV, TB and malaria, HIV is subsumed within one of 169 targets and 17 goals that comprise the SDGs 6. This could be construed as a dilution of attention to HIV, or conversely a redressing of the balance, given the evolution of the global burden of disease and the plurality of major disease threats, especially the rise of non‐communicable diseases (NCDs). The shift from exceptionalism to active integration of HIV across health, education, violence prevention, poverty and the lived environment is the new imperative.

Should the SDGs be framed as a major “threat” to HIV funding, support and response capacity? The SDGs are here, and the HIV community, both epistemic and activist, needs to resituate itself within this new dominant development framework. UNAIDS and the International AIDS Society have spearheaded a conversation and strategic thinking on this reconfigured relationship, capitalizing on the growing sub‐discipline that aims to articulate the “learning from AIDS” to inform and promote solutions to address other public health challenges. These are characterized by complex causal networks, “combination” structures, and the need for advanced investment and governance models which coordinate a spectrum of responding actors across systems and society as a whole 7.

SDG Target 3.3 urges the global community to “By 2030, end the epidemics of AIDS, tuberculosis, malaria and neglected tropical diseases and combat hepatitis, water‐borne diseases and other communicable diseases.” The indicator 3.3.1 is one of five for this target, tracking the “Number of new HIV infections per 1000 uninfected population, by sex, age and key populations.” The scale of intention has also shifted from “halt and reverse” HIV in relation to the previous MDG 6, to the more substantial “end the epidemic” by 2030. While the development scope has broadened, the level of HIV response required has actually intensified.

The complexity of Agenda 2030 entails the need to prioritize and inevitably embark on a series of trade‐offs at country level. The UN Development Group, led by UNDP, is undertaking a series of inter‐agency missions to countries to help develop SDG roadmaps at national and where appropriate, sub‐national level 8. Furthermore, a recent Global Review of the UNAIDS Operational Model recommended introduction of country resource envelopes to domesticate and enhance the joint UN response in priority high HIV burden countries, so called fast‐track countries. This domestication and prioritization of SDGs aims to seek the most cost‐effective combination of investments across the targets, bespoke to the situation and prioritizing interventions that at scale will deliver against multiple targets and goals simultaneously, the “accelerators” of sustainable development. Addressing the causes and consequences of HIV is arguably one of these accelerators.

HIV/AIDS experience has major value in informing the SDG core approaches, over and above being a component response in its own right. UNAIDS argues that “…lessons learned from the multisectoral, multistakeholder AIDS response, including engagement of civil society are key to progress across the SDGs. The AIDS response has advanced such issues as the right to health, gender equality, health information systems, service delivery platforms, commodity access and security and social protection. The response has garnered substantial experience in addressing entrenched social norms, social exclusion and legal barriers that undermine health and development outcomes, and its investment approach is increasingly being adopted to accelerate gains across global health and development. The AIDS response can be a leader in leveraging strategic intersections with the SDGs, while disseminating lessons learned from three decades of unprecedented progress.” 9

The implication is that the HIV response has not only intrinsic value in its efforts to end AIDS by 2030 (as a component of target 3.3), but also has extrinsic value in leveraging other associated and potentially connected development objectives. This framing of the HIV “dividend” relates to both the synergies between HIV response and other objectives, as well as the understanding that the HIV response infrastructure, with its inbuilt experience and precedents, is a significant asset on which to build comprehensive health and social service provision. HIV responses are a driver of universal health coverage, for both integrated service delivery and through deliberate exposure and overcoming of barriers to accessing quality health services. All the while, the human rights of HIV‐affected communities have permeated the advocacy, programme design, monitoring and impact assessment. Action on HIV has deliberately targeted “those left behind”, the core group of obligation in the 2030 Agenda.

HIV‐focused or invested institutions cannot be seen as the sole claimant of this positive scenario. A conversation is ongoing across a broad range of themes regarding their valued contribution, given the synergistic nature of the SDGs and the need to view targets and their associated themes as integral to a larger whole, interdependent on shared successes across targets. We stress, however, that the claim of HIV responses to a central SDG role is a strong one. For example in the field of global health, HIV has innovated through the building and use of evidence, and normalized this research evidence as fundamental to the validity and expansion of the response infrastructure. Understanding this value of the HIV response for children and adolescents within and for the development agenda is the purpose of commissioning this Special Issue, led by the Collaborative Initiative for Paediatric HIV Education and Research at the International AIDS Society.

These papers identify a set of opportunities for addressing paediatric and adolescent HIV that are offered by the SDGs. They highlight the areas of common interest and specific connections with targets and indicators beyond HIV and health alone. They illustrate how HIV and AIDS responses have the latent potential to be a driver across the development arena. Extraordinary knowledge, skills and expertise have been built, new ways of working, of implementation and of embedding evidence‐based practice. Over the past 30 years, the evolution and multiple reinvention of programming has been in response to positive and negative changes such as advances in prevention technology, new treatment regimens and the threat of viral resistance, all with their own intended and unintended consequences. So this is the new challenge, to evolve in a way that capitalizes on the opportunities of the SDGs and that ensures that children and adolescents both vulnerable to and affected by HIV are themselves not left behind.

The papers in this Special Issue address this challenge from a range of perspectives. Several retain the core focus on SDG 3 (healthy lives and wellbeing) and examine new challenges and solutions in the SDG era. All seek synergies at various levels with other SDGs and targets.

Cluver and colleagues demonstrate the impressive effects of combined service provision, as a proxy of SDG interaction, on HIV mortality in adolescents in South Africa. Longevity and survival is associated not only with ART but with food security, social protection and access to non‐HIV health services 10. Chaudhury and colleagues delve deeper into questions of equity of access to treatments and care for children in Tanzania across age groups. Compared to paediatric cases, adolescence is associated with risk of late presentation, delayed treatment initiation and loss of continuity of care. Improving health and wellbeing for all, “at all ages” as required by Goal 3, entails particular attention to the adolescents living with HIV 11. Fatti and colleagues examine treatment adherence and outcomes (notably the degree of viral suppression) for adolescents living with HIV in South Africa, and their findings suggest that community‐based support could be the crucial link between clinical support and progress toward several health, economic and equity‐related SDG targets 12. Slogrove and colleagues assess treatment outcomes in adolescents living with perinatally acquired HIV across 25 countries. They conclude that at the macro‐level and irrespective of ART access, measurable differences in mortality relate to the income status of the country, with poorest outcomes in the lower income countries, highlighting the relevance of promoting equality within and between nations 13.

Kilburn and colleagues generate further evidence that poverty alleviation and reduction of HIV risks are highly connected. In South Africa, conditional cash transfers work in part through delaying sexual debut or reducing the number of sexual partners of adolescent girls and young women. Intimate partner violence is reduced and proves a critical mediator in reducing HIV risks 14. Grosso et al. draw the link between HIV‐related risk behaviours in female sex workers in Lesotho that are directly influenced by experience of sexual abuse as children. The complex causal pathways between child sexual abuse, sex work and HIV risk are mapped out and demand our attention with regard to intervention design and impact mitigation, again with the life course and range of SDGs in full view 15.

Leaving no one behind means full accountability of the state to those who are in need of quality and accessible HIV services. Gleeson and colleagues highlight the need for adolescents and young people to be meaningfully engaged as leaders of HIV within the SDG response 16. Hodes and colleagues examine what happens when this is enacted through participatory engagement. Adolescents in South Africa identified strong needs and linkages between SDGs 2 (end hunger), 3 (healthy lives and wellbeing) and 6 (clean water and sanitation) in material, not abstract policy terms 17.

The SDGs demand policy coherence across development policy domains and innovative partnerships to advance them. Penazzato and colleagues describe how capacity‐building and South‐North collaborations have the potential to accelerate availability of optimized treatment options not only for infectious diseases including HIV, but also for tuberculosis and viral hepatitis, which affect children in low‐ and middle‐income countries 18. Tinasti assesses the criminalization of drug use and punitive policy environments and their impact on adolescents’ health and HIV transmission risks 19. Similarly, Chamla and colleagues explore the connections between HIV and humanitarian setting engagement. With 9 of the 21 countries in Africa deemed by UNAIDS as being “high priority” for HIV, being fragile, conflict‐affected, or impacted by climate‐related hazards, to what extent is HIV and crisis prevention and recovery integrated? While clearly work in progress, the drug policy‐HIV service synergies, and the humanitarian‐development nexus provide important frameworks that could bridge divides between these all‐too‐often disparate areas of concern 20.

We are privileged to have been guest editors for this Special Issue and emerge convinced that while HIV responses were advanced prior to the SDG agenda, ending AIDS will only be possible as one of its core components.

## Competing interests

The authors declare no competing interests.

## Authors’ contributions

DW, LC and CW reviewed all articles in the special issue, and formulated, drafted and developed the editorial. All authors have read and approved the final version.

## References

[1] UNICEF . Children and AIDS: statistical update. New York: UNICEF; 2017.

[2] UNAIDS . Fast‐track ‐ ending the AIDS epidemic by 2030. Geneva: UNAIDS; 2014.

[3] Sherr L , Cluver L , Tomlinson M , Coovadia H , Coalition for Children Affected by AIDS. Defeating AIDS but missing children. Lancet. 2015;386(9998):1035. 10.1016/S0140-6736(15)00134-8 26382987

[4] UNAIDS . UNAIDS data 2017. Geneva: UNAIDS; 2017.

[5] UNICEF . Children and AIDS: statistical update 2017. Johannesburg: UNICEF; 2017.

[6] United Nations . Transforming our world: The 2030 agenda for Sustainable Development. A/RES/70/1: The United Nations; 2016.

[7] Beyrer C , Das P , Horton R , Ryan O , Bekker LG . The International AIDS Society‐Lancet Commission on the Future of the HIV Response and Global Health. Lancet. 2017;390(10092):344–5. 28745592 10.1016/S0140-6736(17)31874-3 PMC6754741

[8] United Nations Development Group . MAPS ‐ mainstreaming, acceleration and policy support for the 2030 Agenda. New York: UNDG; 2015.

[9] UNAIDS . The AIDS response in the 2030 agenda for Sustainable Development: Joint work, shared gains. Available from: http://www.unaids.org/en/AIDS_SDGs: UNAIDS; 2016.(accessed date: 22 November 2017)

[10] Cluver L , Pantelic M , Orkin FM , Toska E , Medley S , Sherr L . Sustainable survival for adolescents living with HIV: Do SDG‐aligned provisions reduce potential mortality risk? J Int AIDS Soc. 2018;21 Suppl 1:e25056. 10.1002/jia2.25056 PMC5978664 29485739

[11] Chaudhury et al Equity of child and adolescent treatment, continuity of care and mortality, according to age and gender among enrollees in a large HIV program in Tanzania. J Int AIDS Soc. 2018;21 Suppl 1:e25070. 10.1002/jia2.25070 PMC5978660 29485735

[12] Fatti G , et al. The effectiveness and cost‐effectiveness of community‐based support for adolescents receiving antiretroviral treatment: an operational research study in South Africa. J Int AIDS Soc. 2018;21 Suppl 1:e25041. 10.1002/jia2.25041 PMC5978711 29485714

[13] Slogrove AL , et al. Inequality in outcomes for adolescents living with perinatally‐acquired HIV in sub‐Saharan Africa: a Collaborative Initiative for Paediatric HIV Education and Research (CIPHER) Cohort Collaboration analysis. J Int AIDS Soc. 2018;21 Suppl 1:e25044. 10.1002/jia2.25044 PMC5978669 29485724

[14] Kilburn K , et al. CCTs and the reduction of partner violence for young women: An investigation of causal pathways using evidence from a randomized experiment in South Africa (HPTN 068). J Int AIDS Soc. 2018;21 Suppl 1:e25043. 10.1002/jia2.25043 PMC5978692 29485746

[15] Grosso A , et al. HIV risks and needs related to the Sustainable Development Goals among female sex workers who were commercially sexually exploited as children in Lesotho. J Int AIDS Soc. 2018;21 Suppl 1:e25042. 10.1002/jia2.25042 PMC5978702 29485709

[16] Gleeson H , et al. Ending AIDS by 2030: the importance of an interlinked approach and meaningful youth leadership. J Int AIDS Soc. 2018;21 Suppl 1:e25061. 10.1002/jia2.25061 PMC5978642 29485749

[17] Hodes R . The stuff that dreams are made of: using participatory research to explore interlinkages in HIV‐positive adolescents’ aspirations for development. J Int AIDS Soc. 2018;21 Suppl 1:e25057. 10.1002/jia2.25057 PMC5978641 29485764

[18] Penazzato M , et al. Shortening the decade‐long gap between having optimal adult and paediatric drug formulations – A new framework based on the HIV experience in low‐ and middle‐income countries. J Int AIDS Soc. 2018;21 Suppl 1:e25049. 10.1002/jia2.25049 PMC5978668 29485727

[19] Tinasti K . HIV and AIDS among adolescents who use drugs: opportunities for drug policy reform within the sustainable development agenda. J Int AIDS Soc. 2018;21 Suppl 1:e25045. 10.1002/jia2.25045 PMC5978662 29485748

[20] Chamla D , et al. Children, HIV, emergencies, and sustainable development goals: roadblocks ahead and possible solutions. J Int AIDS Soc. 2018;21 Suppl 1:e25046. 10.1002/jia2.25046 PMC5978666 29485728

